# Luminance Matching in Cognitive Pupillometry Is Not Enough: The Curious Case of Orientation

**DOI:** 10.1523/ENEURO.0238-25.2025

**Published:** 2025-11-05

**Authors:** Matthew A. Parrella, Isshori Gurung, Michael A. Grubb

**Affiliations:** Trinity College, Hartford, Connecticut 06106

**Keywords:** expectation, pupillometry

## Abstract

Abrupt onsets reflexively shift covert spatial attention. Recent work demonstrated that trial-to-trial information about the probability of a peripheral onset modulated the magnitude of the attentional cueing effect (low probability > high probability). Although onsets were physically identical, pupil responses could have been modulated by information about the probability of the onset's appearance. Specifically, anticipatory constrictions may have preceded high-probability onsets. Here, we tested this hypothesis using centrally presented, luminance-matched onset-probability signals. For half the participants, vertical signaled high probability (0.8) of onset appearance, while horizontal signaled low probability (0.2). Contingencies were reversed for the other half. Participants fixated the onset-probability signal for 2,000 ms before the onset was briefly presented or omitted, in line with the signaled probability. To maintain engagement, participants completed a simple localization task. Preliminary evidence for an anticipatory reduction in the pupil area was obtained in Experiment 1. However, this effect disappeared in Experiment 2 with a larger replication sample. Exploratory analyses uncovered a violation of a fundamental methodological assumption: despite being task-irrelevant and perfectly luminance-matched, vertical onset-probability signals consistently generated smaller pupil areas, relative to horizontal signals in both Experiments 1 and 2. Interestingly, this “orientation effect” was stronger in the second half of the experimental session, and in a third experiment, we significantly reduced its magnitude by changing the locations of the task-relevant stimuli. In short, across three experiments (self-reported gender, 52 females, 26 males, 1 nonbinary), we show that even with perfect luminance matching, unforeseen changes in cognitive state can modulate pupillometric measurements.

## Significance Statement

Cognitive pupillometry often relies on the assumption that matching stimuli for luminance will control for unwanted influences on the pupil area. Here, we demonstrate that a simple, task-irrelevant white line—identical in luminance, size, and spatial location—elicited systematically smaller pupil areas when oriented vertically rather than horizontally. This orientation effect was present in two independent datasets; it emerged with task experience, and it was attenuated by changing the locations of task-relevant stimuli. Our findings reveal an unexpected, and potentially hidden, confound in cognitive pupillometry. In light of the pupil's remarkable sensitivity, researchers should carefully consider whether stimulus features beyond luminance—such as orientation and its interaction with broader experimental design choices—inadvertently affect pupil-based measures of cognitive processes.

## Introduction

Recently published research has shown that centrally presented, symbolic information about the prior probability that a task-irrelevant stimulus will briefly flash in the visual periphery modulates the degree to which peripheral onsets drive the reflexive allocation of spatial attention (Crotty et al., 2025). In their study, Crotty and colleagues manipulated the probability that an exogenous, peripheral onset would appear or not appear on a given trial, and this probability was communicated at the beginning of the trial using a color-defined signal at fixation (e.g., green signaled a high onset-probability of 0.8; red signaled a low onset-probability of 0.2, with color-probability contingencies counterbalanced across participants). When the signal indicated that the probability of the peripheral onset was low but it appeared anyway, the onset elicited a stronger cueing effect (change in task accuracy, valid vs invalid cues) than an identical onset presented after a signal indicating a high onset-probability. This investigation provided preliminary psychophysical evidence of an interaction between expectation and the reflexive allocation of covert spatial attention, adding to a growing literature on the role of expectation in visual perception more broadly (Summerfield and Egner, 2009; Summerfield and de Lange, 2014; de Lange et al., 2018; Press et al., 2020).

Crotty and colleagues were careful to manipulate spatial attention using the methodological “best practices” in the field (Carrasco, 2011). Orientation discrimination judgments were made for the same grating stimuli, presented at the same peripheral locations, following the presentation of a spatially uninformative, peripheral onset which was identical in both valid and invalid trials and preceded the stimulus array with an appropriate stimulus-onset asynchrony of 100 ms. Inspired by predictive-processing accounts of perception, we hypothesized that expectations about a sudden flash in the visual periphery may have induced anticipatory changes in the pupil area, which would create an asymmetry in the pupil area on high versus low onset-probability trials in that study. If such an anticipatory constriction could be verified experimentally, it would provide a more nuanced understanding of the results reported by Crotty et al. (2025).

**Figure 1.**
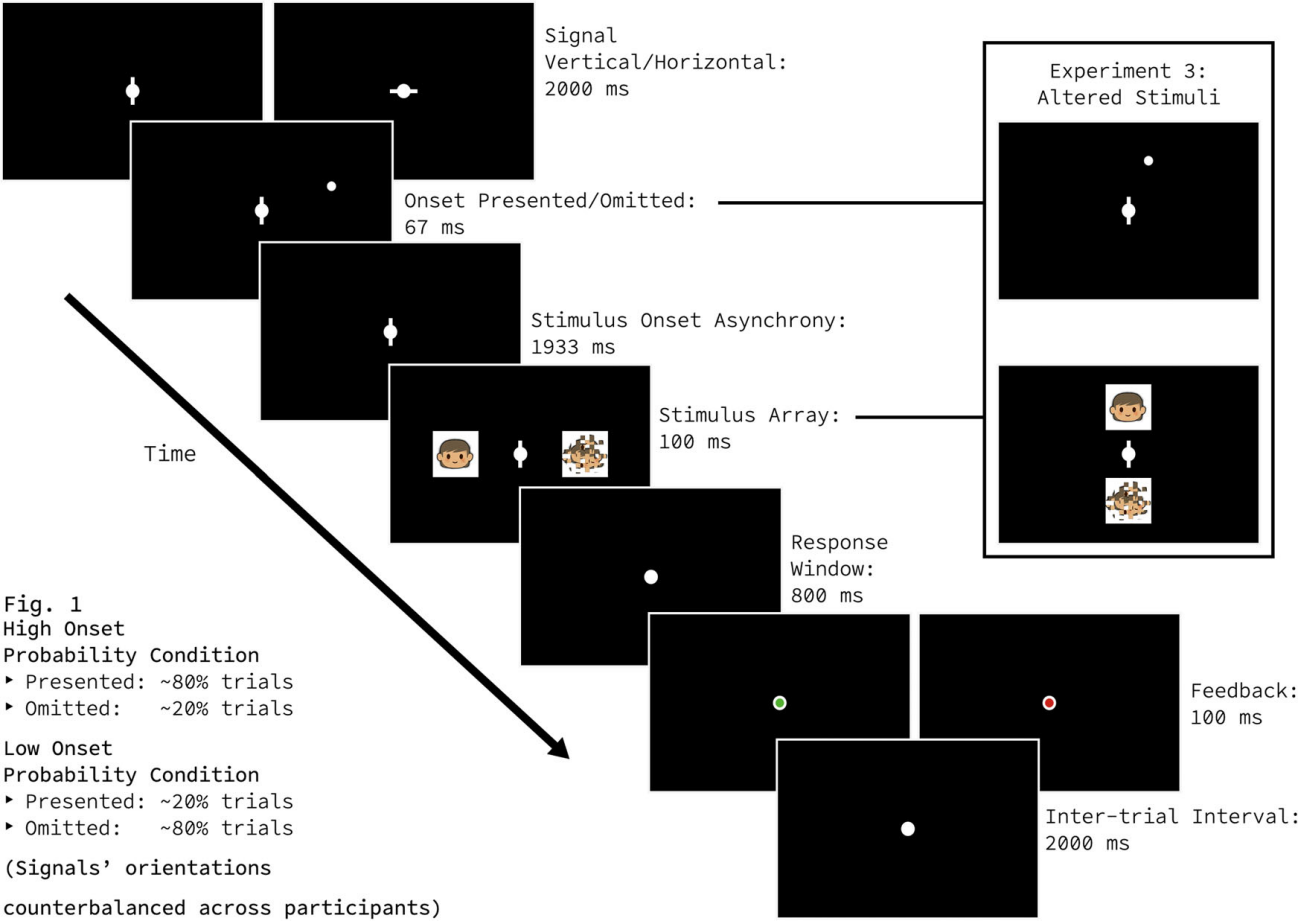
Trial sequence of Experiments 1 and 2. Experiment 3 used the same stimuli and trial timing, but the stimulus array was presented along the vertical meridian.

Here, we leveraged the methodological insights provided by Mathôt and Vilotijević (2023) and designed an experiment to test this hypothesis (Fig. 1). At the start of each trial, we signaled that a peripheral onset was likely or unlikely by associating the orientation of a centrally presented line with different probabilistic outcomes. For half of the participants, vertical signals indicated high onset-probability (0.8), whereas horizontal signals indicated low onset-probability (0.2); contingencies were reversed for the other half of the participants. To keep participants engaged, they completed a simple target localization task 4,000 ms after the presentation of the onset-probability signal. As in Crotty and colleagues’ 2025 study, neither the onset-probability signal nor the location of the abrupt, peripheral onset provided any task-relevant information; participants were told so explicitly and were instructed to ignore the line at fixation and any flashes that may or may not appear in the periphery. In short, the pupil area was measured in a slow-paced experiment, while participants did nothing but fixate on the same constant stimulus (the onset-probability signal) at the same central location. We took seriously the recommendation that “[s]timuli should ideally be constant between conditions,” so as to be in line with the “Hillyard principle” (Mathôt and Vilotijević, 2023, p. 3058). Rather than use a color-defined signal as in the Crotty et al. (2025) study, here we used an onset-probability signal that was not only luminance-matched but was physically identical in all respects except orientation. We reasoned that orientation would be a good choice because two distinct orientations will generate two distinct patterns of neural activity in early visual areas (Kamitani and Tong, 2005), which could serve as strong, internally generated signals for implicit learning.

To preview our results, we found that a fundamental methodological assumption did not hold in our experimental protocol: despite being perfectly matched for luminance, size, and spatial location—and entirely task-irrelevant—the pupil responded differently to vertical, relative to horizontal, signals in our first two experiments. Data from a third experiment confirmed that this “orientation effect” was contingent on the spatial configuration of the task-relevant locations. Taken together, these findings highlight a strength of the pupillometric method (exquisite sensitivity to subtle features of the task structure) and provide an important reminder that even with perfect luminance matching, unanticipated changes in cognitive state can modulate pupillometric measurements.

## Materials and Methods

### Participants

The study was approved by the local Institutional Review Board (IRB), and all methods were performed in accordance with the relevant IRB guidelines and regulations. Prior to all sessions, participants provided written informed consent.

#### Experiment 1

Fifteen participants (nine females and six males; 19–30 years old; mean, 20.36) completed one session (400 trials each).

#### Experiment 2

Thirty-nine participants (24 females and 15 males; age 18–46; mean, 20.36) completed one session (400 trials each).

#### Experiment 3

Twenty-five participants (19 females, 5 males, 1 nonbinary; 18–29 years old; mean, 19.6) completed one session (400 trials each).

### Compensation

Participants received $20.00 for the ∼1 h session.

### Instructional video

Participants watched the instructional video available here: www.attentionPerceptionDecision.com/PGG_eNeuro.

### Apparatus

The perceptual task was programmed in PsychoPy (Peirce et al., 2019) and run on a 3.2 GHz 6-Core Intel Core i7 Mac Mini; stimuli were displayed on a 27.0″ LED-Lit Dell Gaming Monitor (model, S2716DG), with a screen resolution of 2,560 × 1,440 pixels. Participants were positioned at a chin-rest ∼70 cm from the monitor. The pupil area was recorded with an EyeLink 1000 Plus (SR Research) at 500 HZ. Calibration was performed with a white nine-point grid on a black background. Participants indicated their response via an Apple A1243 Keyboard.

### Calibration

At the beginning of the session, the eye tracker was calibrated for the first block. There were 8 blocks throughout the session, each with 50 trials. Between blocks, participants were allowed to take a break, exit the eye-tracker apparatus to rest, and return for recalibration before the next block.

### Experimental manipulation

Throughout each block, participants held their gaze at the fixation circle and were instructed to ignore two elements. First, at the beginning of the trial, a line appeared at fixation, where it remained until the task stimuli disappeared. Second, a small white circle, or “onset,” had a 50% chance of flashing in the upper-right periphery. The probability of onset appearance in a given trial was indicated by the orientation of the line at fixation (the “onset-probability signal”). For half of the participants, a vertical onset-probability signal indicated that the peripheral onset would appear with probability 0.8, and a horizontal onset-probability signal indicated that the peripheral onset would appear with probability 0.2. The association between orientation and onset-probability was reversed for the other half of participants.

### Task

Participants performed a two-alternative, forced-choice localization task. In Experiments 1 and 2, the left and right arrow keys were used to indicate which side of the display (left or right) contained an intact emoji image. In Experiment 3, the up and down arrow keys were used to indicate whether the intact emoji image was above or below fixation.

### Experimental stimuli

#### Fixation circle

The fixation circle was white, with a radius of 0.25° of the visual angle (d.v.a.). It appeared at the center of the screen through the entirety of each 5,000 ms trial and between each 2,000 ms intertrial interval.

#### Onset-probability signal

The onset-probability signal was a narrow, white rectangle. In its vertical orientation, the height of the onset-probability signal was 1 d.v.a., and its width was 0.1 d.v.a.; in its horizontal orientation, the height and width of the onset-probability signal were reversed. The onset-probability signal appeared at (0 d.v.a., 0 d.v.a.), overlaying the fixation circle for 4,100 ms at the beginning of the trial; its offset was coincident with the offset of the task stimuli (see below).

#### Peripheral onset

The peripheral onset was a white circle with a radius of 0.25 d.v.a. When presented, it appeared 2,000 ms after the onset-probability signal in the right visual periphery (6 d.v.a., 2.5 d.v.a. in Experiments 1 and 2; 2.5 d.v.a., 6 d.v.a. in Experiment 3) and disappeared after 67 ms.

#### Task stimuli

Two images simultaneously appeared 4,000 ms after the onset-probability signal, one to the left and one to the right of the central fixation circle (±6 d.v.a., 0 d.v.a.) in Experiments 1 and 2 and one above and below the central fixation circle (0 d.v.a., ±6 d.v.a.) in Experiment 3. Both images disappeared after 100 ms. In a given trial, one image depicted an Apple emoji of a person on a white background; the other was a scrambled version of the same image. Both stimuli were square images with a height and width of 250 pixels, which corresponded to ∼4.78 d.v.a. in our setup.

#### Task stimuli opacity

At the beginning of the session, the opacity of the images was set to 1. This opacity value was modified via a one-up/two-down staircase procedure in which we halved the opacity after two correct responses and doubled the opacity after one incorrect response. Opacity values were constrained to not fall below 0.008 or above 1.

#### Feedback circle

The feedback circle was identical to the fixation circle but was filled with either green, following a correct response, or red, following an incorrect response. After the 800 ms response window, feedback was displayed for 100 ms.

### Data preprocessing

**Figure 2.**
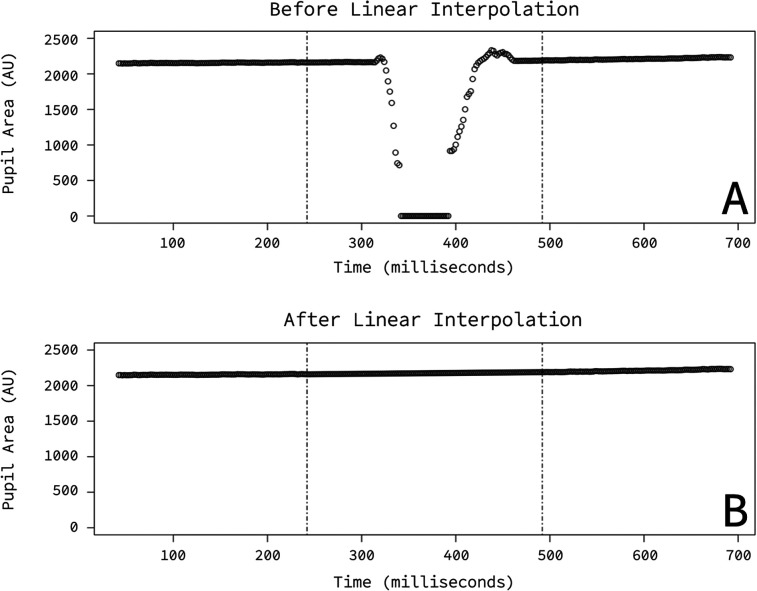
Blink interpolation. ***A***, Example pupil area during a blink. ***B***, Example pupil area after blink interpolation.

Pupil samples during blinks were identified by EyeLink's built-in blink detection algorithm and then transformed by linear interpolation. A 100 ms pad before and after blink intervals defined the interpolation window (marked by dashed lines in Fig. 2*A*,*B*). Linear regression was used to interpolate between the first and last sample of this window. Blinks occurred during 4.9279% of the 2,000 ms onset-probability signal window in Experiment 1, during 8.5506% of the 2,000 ms onset-probability signal window in Experiment 2, and during 8.5504% of the 2,000 ms onset-probability signal window in Experiment 3. Any remaining samples in which the pupil area was zero were replaced with NA.

Subtractive baseline correction (Mathôt et al., 2018) was performed using interpolated pupil samples. For each trial, the 20 ms preceding the appearance of the onset-signal were selected as the trial's baseline window. The median pupil sample from this window was subtracted from all samples in the trial.

### Data availability

Upon publication, all data and analysis scripts will be available from the corresponding author's website: www.attentionPerceptionDecision.com/PGG_eNeuro.

## Results

### Experiment 1: the impact of onset-probability information on the pupil area

**Figure 3.**
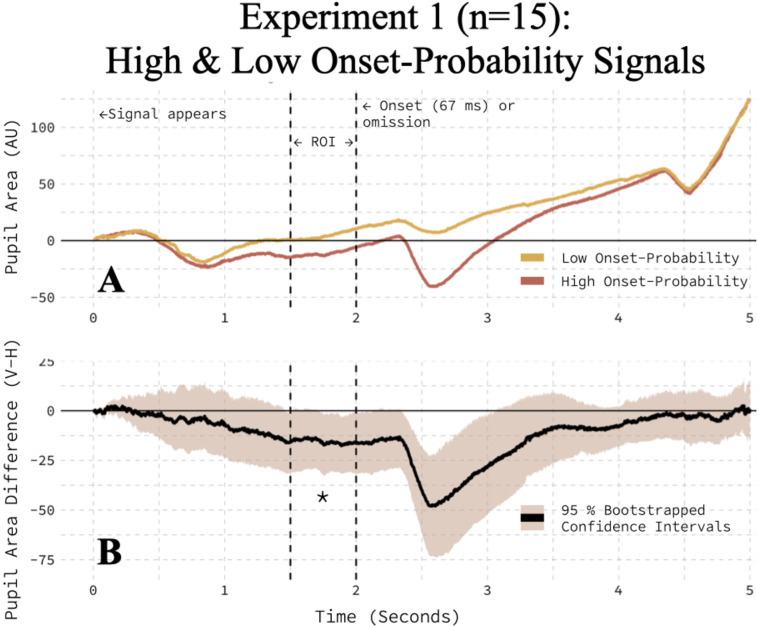
Onset-probability modulated the pupil area in Experiment 1. ***A***, The pupil area was smaller on high onset-probability than low onset-probability trials. ***B***, The average, within-participant difference between high and low onset-probability conditions is bootstrapped with a 95% confidence interval.

The pupil area was smaller when the onset-probability signal indicated high, relative to low, onset-probability in a preliminary sample of 15 participants. For each observer, we grouped trials by onset-probability condition, aligned pupil data to the appearance of the signal, and computed the average pupil trace, with all values expressed relative to a presignal baseline computed via subtractive baseline correction. To give participants time to implicitly learn the contingencies, we restricted this analysis to the session's second half (trials 201–400). Figure 3*A* shows each condition, averaged across participants. Within-participant differences were computed (high minus low onset-probability), and a bootstrapped 95% confidence interval was obtained by randomly sampling individual participants with replacement (Fig. 3*B*). We defined an arbitrary, temporal region of interest (ROI) 500 ms before onset presentation or omission (Fig. 3*A*,*B*, dashed lines) and computed the average, within-participant difference in the pupil area. A paired *t* test revealed that the pupil area was reduced when the onset-probability signal indicated that a peripheral cue was likely, relative to unlikely (*t*_(14)_ = −1.97; *p* = 0.0343, one-tailed in predicted direction). Onset-probability signals differed only in orientation and were matched for luminance, size, and spatial location. Thus, we attributed these findings to the signal's probabilistic information. Having found preliminary evidence in support of our hypothesis, we ran a replication study with more participants to confirm the reliability of this anticipatory reduction in the pupil area and further assess rare events (e.g., the pupil response to low-probability onsets, which occur only in ∼10% of trials).

### Experiment 2: the impact of onset-probability information on the pupil area

**Figure 4.**
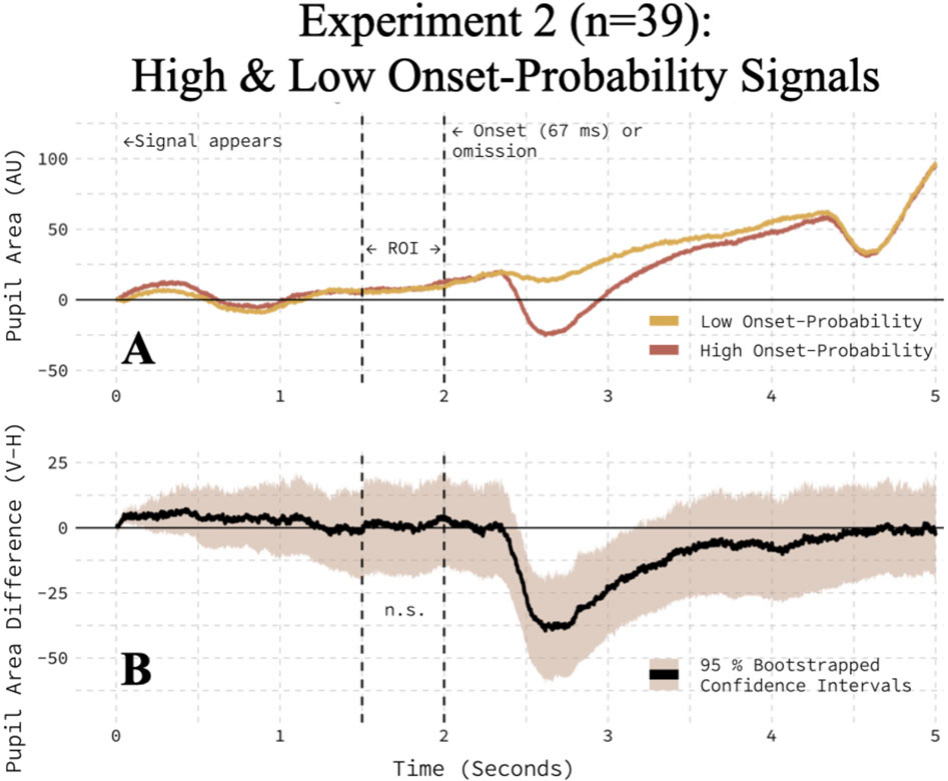
Onset-probability did not modulate the pupil area in Experiment 2. ***A***, The pupil responded similarly to the high and low onset-probability signals. ***B***, The average, within-participant difference between high and low onset-probability conditions is bootstrapped with a 95% confidence interval.

The onset-probability-induced, anticipatory relative reduction in the pupil area observed in Experiment 1 did not replicate in a larger sample of 39 participants. There was no clear difference in the pupil's response during the same ROI (Fig. 4*A*,*B*), and a paired *t* test found no evidence that the pupil area was modulated by the onset-probability signal (*t*_(38)_ = 0.13; *p* = 0.5513; one-tailed in predicted direction). The failure to replicate the results of Experiment 1 in Experiment 2 was puzzling and prompted us to explore the data further.

### Experiments 1 and 2: the impact of the onset-probability signal’s orientation on the pupil area

**Figure 5.**
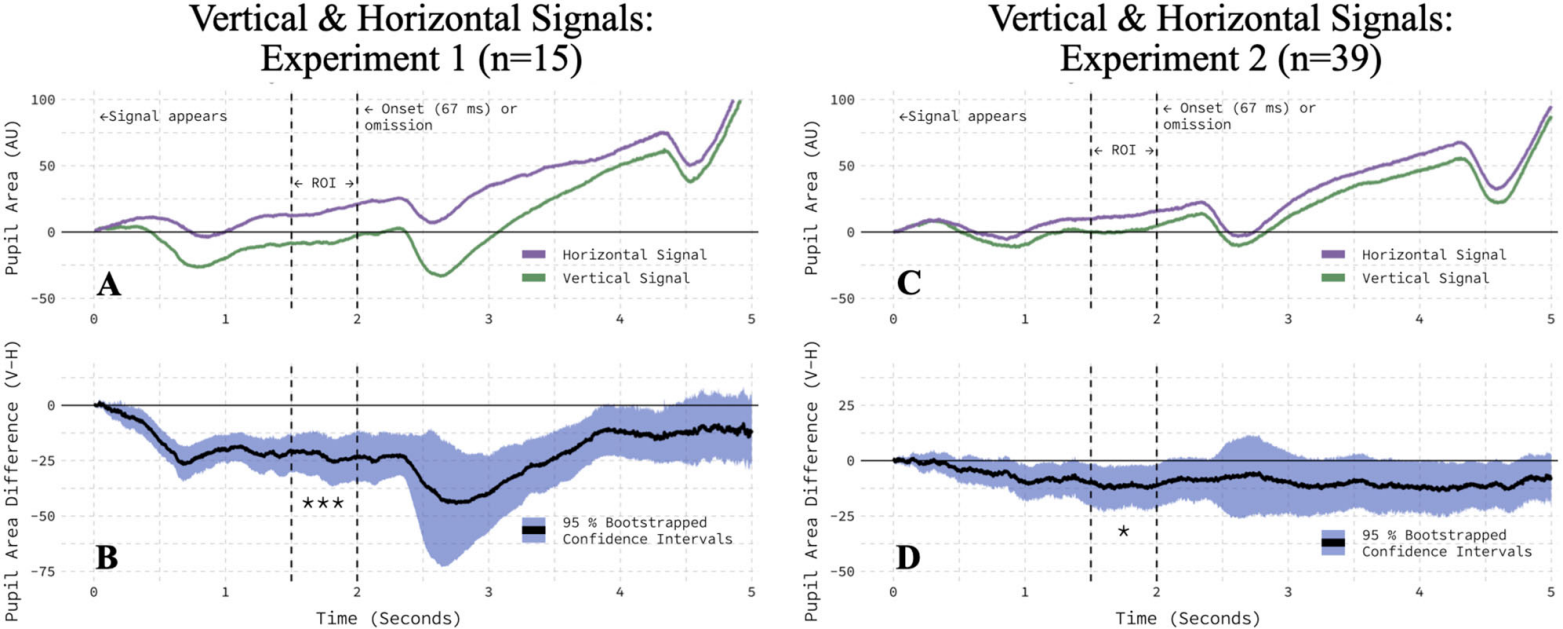
Orientation modulated the pupil area in both experiments. ***A***, The pupil area was smaller on vertical than horizontal onset-probability signal trials in Experiment 1. ***B***, The average, within-participant difference between vertical and horizontal conditions is bootstrapped with a 95% confidence interval. ***C***, The pupil area was smaller on vertical than horizontal onset-probability signal trials in Experiment 2. ***D***, The average, within-participant difference between vertical and horizontal conditions is bootstrapped with a 95% confidence interval.

An analysis of all trials in the session uncovered a violation of a fundamental methodological assumption: vertical onset-probability signals engendered smaller pupil areas than horizontal signals. This pattern of results was observed independently in both experimental samples (Fig. 5*A*,*C*). Within our 500 ms ROI, bootstrapped 95% CIs excluded zero (Fig. 5*B*,*D*), and two-tailed, paired *t* tests confirmed a significant difference in the pupil area (Experiment 1, *t*_(14)_ = −4.26; *p* = 0.0008; Experiment 2, *t*_(38)_ = −2.38; *p* = 0.0223). To verify that this effect did not depend on our statistical approach or our arbitrarily chosen ROI and to alleviate any potential concerns about multiple comparisons, we applied the trial-level cross–validation procedure of Mathôt and Vilotijević (2023) to the final 1,500 ms of the 2,000 ms onset-probability signal window (Fig. 1, first panel). This window allows 500 ms for any modulation of the pupil light response to emerge and ends before the presentation of the peripheral onset (in trials that have one), which will drive its own change in the pupil area. Once again, vertical signals produced smaller pupil areas than horizontal in both experimental groups (Table 1, Rows 1–2). In short, although matched for luminance, size, and spatial location—and entirely task-irrelevant—vertical onset-probability signals led to smaller pupil areas than horizontal signals.

**Table 1.** Trial-level cross–validation analyses using the Python library time_series_test, as presented and discussed in Mathôt and Vilotijević (2023)

	Data assessed	Sample size	Model	Statistics	Samples tested
1	Experiment 1 (E1)	*n* = 15	pupil ∼ isVertical (isV)	*z* = −5.053, ***p* < 0.0001**	688, 712, 732 ms
2	Experiment 2 (E2)	*n* = 39	pupil ∼ isV	*z* = −2.040, ***p* = 0.0413**	1,024, 1,596, 1,676, 1,960 ms
3	Vertical = high onset-probability, E1 and E2	*n* = 27	pupil ∼ isV	*z* = −2.998, ***p* = 0.0027**	1,278, 1,678, 1,842, 1,904 ms
4	Vertical = low onset-probability, E1 and E2	*n* = 27	pupil ∼ isV	*z* = −2.167, ***p* = 0.0302**	680, 792, 822, 954 ms
5	E1 and E2	*n* = 54	pupil ∼ isV*verticalHighProb	*z* = 0.093, *p* = 0.9256	500, 1,306, 1,464, 1,874 ms
6	1st Half, E1 and E2	*n* = 54	pupil ∼ isV	*z* = −0.373, *p* = 0.7093	638, 1,928, 1,942 ms
7	2nd Half, E1 and E2	*n* = 54	pupil ∼ isV	*z* = −3.225, ***p* = 0.0013**	756, 974, 980, 1,730 ms
8	E1 and E2	*n* = 54	pupil ∼ isV*isFirstHalf	*z* = 2.2246, ***p* = 0.02611**	978, 980, 1,608 ms
9	2nd Half, E1/E2 and E3	*n* = 79	pupil ∼ isV*isExp3	*z* = 2.9961, ***p* = 0.0027**	1,676, 1,680, 1,854, 1,998 ms
10	2nd Half, E1/E2 (first 25) and E3	*n* = 50	pupil ∼ isV*isExp3	*z* = 2.6272, ***p* = 0.0086**	936, 974, 1,014, 1,788 ms

All analyses were restricted to the final 1,500 ms of the 2,000 ms onset-probability signal window (Fig. 1, Panel 1). Times in the “Samples tested” column are relative to the appearance of the onset-probability signal.

Bold values indicate statistically significant results.

**Figure 6.**
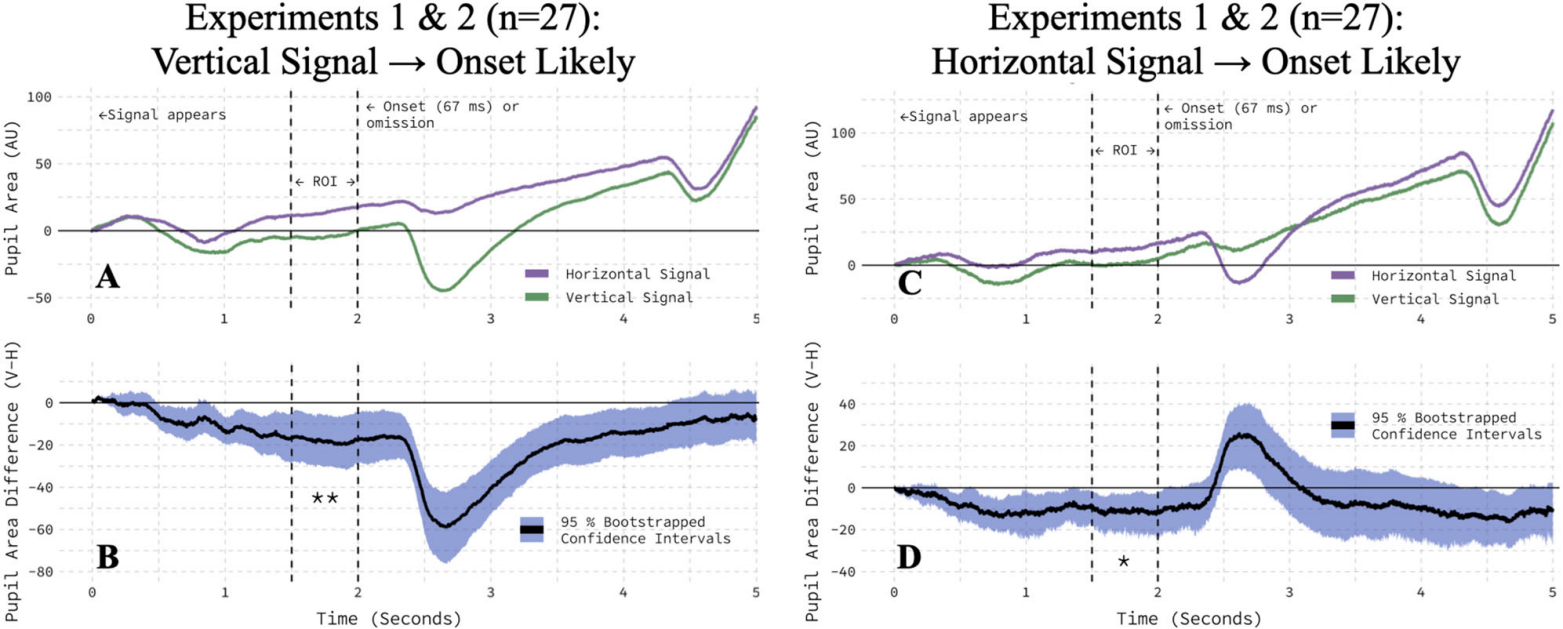
Orientation modulated the pupil area in both cue-onset contingency conditions. ***A***, The pupil area was smaller in response to vertical than horizontal when vertical signaled a high onset-probability. ***B***, The average, within-participant difference between vertical and horizontal conditions is bootstrapped with a 95% confidence interval. ***C***, The pupil area was smaller in response to vertical than horizontal when vertical signaled a low onset-probability. ***D***, The average, within-participant difference between vertical and horizontal conditions is bootstrapped with a 95% confidence interval.

This orientation-dependent modulation of the pupil area was not confounded by onset-probability information. In each experiment, the relationship between the orientation of the onset-probability signal (vertical, horizontal) and information about the probability of an abrupt, peripheral onset appearing in that trial (high, low) was counterbalanced across participants. To rule out the possibility that our results were impacted by these contingencies, we pooled the data from the two experiments and repeated our analysis, separately for participants assigned to each contingency. In both groups of 27 participants, we observed the same pattern: vertical onset-probability signals consistently generated smaller pupil areas than did horizontal signals (Fig. 6*A*,*C*). Within our 500 ms ROI, bootstrapped 95% CIs excluded zero (Fig. 6*B*,*D*), and two-tailed, paired *t* tests confirmed a significant difference in the pupil area (vertical signaled high onset-probability, *t*_(26)_ = −2.97; *p* = 0.0063; vertical signaled low onset-probability, *t*_(26)_ = −2.43; *p* = 0.0225). Furthermore, the two groups did not differ in the size of the effect (Welch two-sample test, *t*_(48.47)_ = −0.90; *p* = 0.3722). Lastly, these results were corroborated by trial-level cross–validation (Table 1, Rows 3–5). In short, onset-probability information did not interact with the impact of the onset-probability signal's orientation on the pupil area.

**Figure 7.**
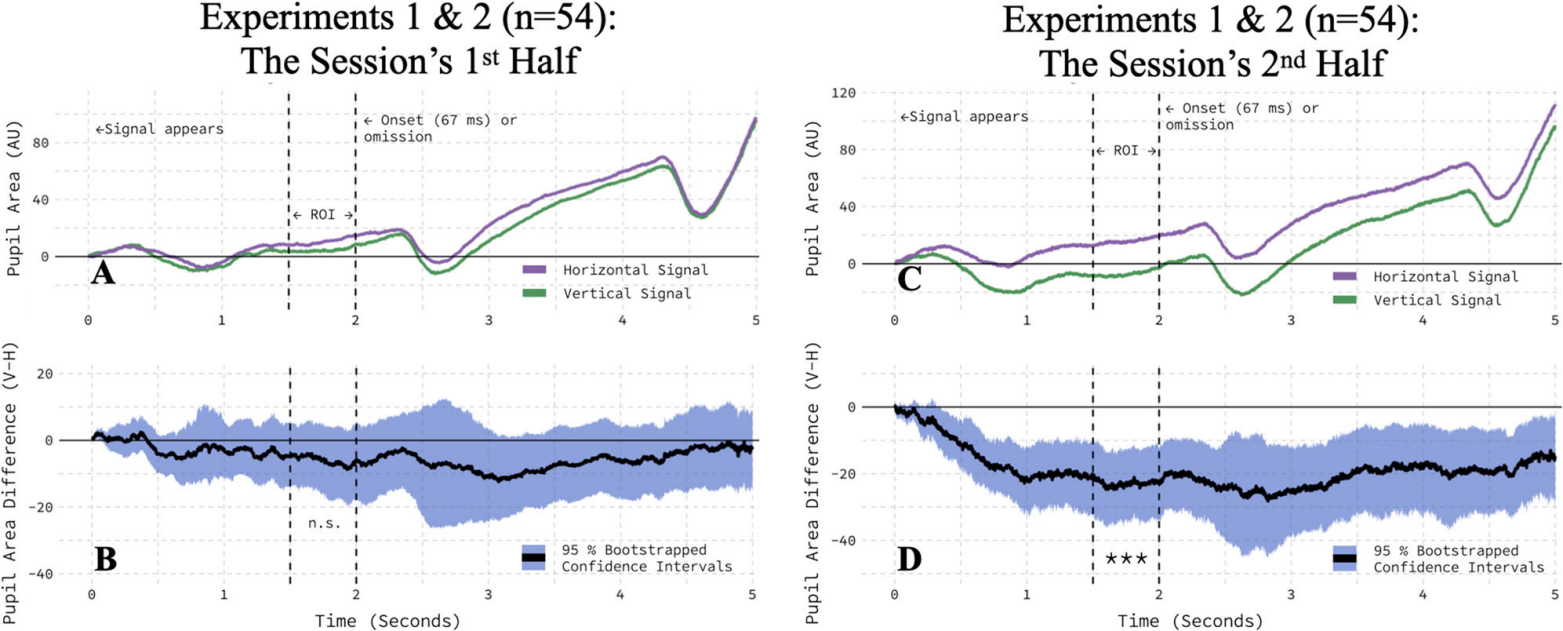
Orientation reliably modulated the pupil area only in the second half of the session. ***A***, The orientation of the onset-probability signal exerted little effect in the first half of the experimental session. ***B***, The average, within-participant difference between vertical and horizontal conditions is bootstrapped with a 95% confidence interval. ***C***, The pupil area was smaller on vertical than horizontal onset-probability signal trials in the second half of the experimental session. ***D***, The average, within-participant difference between vertical and horizontal conditions is bootstrapped with a 95% confidence interval.

Separately assessing the first half of the session (trials 1–200) and the second half (trials 201–400) demonstrated that the impact of the onset-probability signal's orientation on the pupil area required experience with the task. To maximize statistical power, we pooled data from both experiments as in the contingency-based analysis above. In the first half of the session, the pupil responses to vertical and horizontal signals were statistically indistinguishable (Fig. 7*A*). Bootstrapped 95% confidence intervals included zero (Fig. 7*B*), and there was no evidence that orientation modulated the pupil area during the 500 ms ROI (*t*_(53)_ = −1.17; *p* = 0.2485). That said, in the second half of the session, the orientation effect became clear (Fig. 7*C*). Bootstrapped 95% confidence intervals did not include zero (Fig. 7*D*), and a two-tailed, paired *t* test showed that the pupil area was significantly reduced in response to vertical, relative to horizontal, onset-probability signals during the ROI (*t*_(53)_ = −3.88; *p* = 0.0003). Furthermore, the absolute magnitude of the orientation effect in the second half was significantly larger than that observed in the first half (*t*_(53)_ = 2.10; *p* = 0.0410), evidencing an orientation by session-half interaction. These results were corroborated by trial-level cross–validation (Table 1, Rows 6–8).

Small gaze deviations cannot account for the impact of orientation on pupil area observed in Experiments 1 and 2. For each participant and each trial, we computed the mean distance from fixation during the 500 ms ROI; we then averaged these values separately for trials with vertical and horizontal signals. A two-tailed, paired *t* test demonstrated that mean deviation from fixation was smaller when the onset-probability signal was vertical relative to horizontal (*t*_(53)_ = −2.34; *p* = 0.023). That said, this difference cannot explain the effect of the onset-probability signal's orientation on the pupil area. The dataset was median-split according to the extent of participants’ deviation differences, with two-tailed, paired *t* tests revealing statistically significant deviation biases in each group, but in opposing directions (participants’ deviation biases above the median, *t*_(26)_ = 3.87; *p* < 0.001; participants’ deviation biases below the median, *t*_(26)_ = −4.61; *p* < 0.001). Importantly, a two-tailed, paired *t* test confirmed that the orientation effect holds for both groups (participants’ deviation differences above the median, *t*_(26)_ = −2.24; *p* = 0.0337; participants’ deviation differences below the median, *t*_(26)_ = −3.13; *p* = 0.0043). Furthermore, the magnitude of the orientation effect did not differ between the two groups (Welch two-sample *t* test, *t*_(49.73)_ = 1.03; *p* = 0.3089). Lastly, the magnitude of the within-participant orientation effect did not correlate with the within-participant deviation difference (rs = 0.0661; *p* = 0.6342). In short, we can have confidence that small gaze deviation differences do not underlie the pupil results in Experiments 1 and 2.

### Experiment 3: the impact of task locations

**Figure 8.**
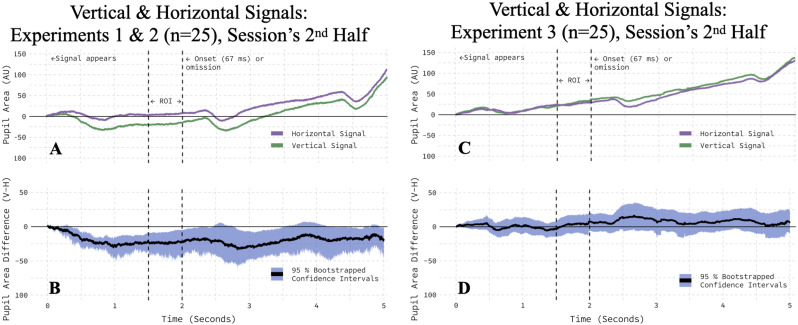
The location of task-relevant stimuli modulates the pupil's orientation response in the second half of the session. ***A***, When task stimuli were arranged left and right of the onset-probability signal, the signal's orientation elicited an orientation-dependent pupillary response in a subset of participants from Experiments 1 and 2. ***B***, The average, within-participant difference between vertical and horizontal conditions is bootstrapped with a 95% confidence interval. ***C***, When task stimuli were arranged above and below the onset-probability signal, the signal's orientation had no effect on the pupillary response. ***D***, The average, within-participant difference between vertical and horizontal conditions is bootstrapped with a 95% confidence interval.

Moving task-relevant stimuli to the vertical meridian in Experiment 3, from the horizontal meridian in Experiments 1 and 2, significantly reduced the impact of the onset-probability signal's orientation on the pupil area. When the target emoji and its scrambled counterpart are presented along the horizontal meridian, as in our first two experiments, a horizontal context signal essentially “points to” the task-relevant locations, whereas a vertical context signal does not. Given the interaction with session-half reported in the previous paragraph, we speculated that this may matter. If it does matter and the target emoji and its scrambled counterpart were moved to the vertical meridian, then we should see a weakening (or complete reversal) of the orientation effect. A new group of 25 participants completed a “vertical layout” version of the experiment (Fig. 8*C*,*D*), and as predicted, the magnitude of the orientation-induced pupil modulation was significantly reduced, relative to the “horizontal layout” version, in the second half of the session (Welch two-sample test, *t*_(58.06)_ = −2.85; *p* = 0.0060). To ensure that the difference in sample size between the two task versions did not confound these results, we compared the first 25 participants who completed the horizontal layout version with the 25 participants who completed the vertical layout version and found the same significant weakening (Welch two-sample test, *t*_(45.77)_ = −2.39; *p* = 0.0211). Both results were corroborated by trial-level cross–validation (Table 1, Rows 9–10).

### Task accuracy

In all experiments, task accuracy was high and unaffected by the orientation of the onset-probability signal (Table 2).

**Table 2.** Task accuracy was high and unaffected by the orientation of the onset-probability signal in all experiments

Experiment	Sample size	Mean proportion correct (SD) vertical	Mean proportion correct (SD) horizontal	Paired *t* test results
1	*n* = 15	0.9747 (0.03)	0.9670 (0.03)	*t*_(14)_ = 1.64, *p* = 0.1234
2	*n* = 39	0.9138 (0.07)	0.9185 (0.06)	*t*_(38)_ = −1.08, *p* = 0.2874
3	*n* = 25	0.933 (0.06)	0.9326 (0.06)	*t*_(24)_ = 0.08, *p* = 0.9394

## Discussion

Here, we designed an experiment to test the hypothesis that information about an impending peripheral onset (a small, white, peripheral circle) induces an anticipatory pupil constriction. The key experimental manipulation involved a centrally presented onset-probability signal: for half the participants, vertical lines at fixation signaled high probability of onset appearance, while horizontal lines signaled low probability (contingencies reversed for the other half). Importantly, the onset-probability signals were perfectly luminance-matched and only differed in orientation. Both the onset-probability signal and the onset itself (should it be presented) provided no task-relevant information. The participants were only asked to maintain fixation and report the location of an intact emoji image presented at one of two possible locations. Data from two experiments uncovered a violation of a fundamental methodological assumption: despite being task-irrelevant, luminance-matched, and presented at fixation, a vertical onset-probability signal consistently generated smaller pupil areas, relative to the presentation of a horizontal signal. Statistical evidence for this orientation-dependent modulation was present in both experiments independently. Although the relative orientation effect was robust, it remains uncertain whether it relates directly to pupillary constriction or dilation in the absence of a neutral baseline condition. Combined analyses confirmed that this effect was not confounded by onset-probability information and that it was stronger in the second half of the session (i.e., it emerged with experience in the task context). A third experiment demonstrated that the alignment of the onset-probability signal's orientation and the task-relevant locations (along the horizontal meridian, Experiments 1 and 2; along the vertical meridian, Experiment 3) modulated the magnitude of this effect.

With respect to the validity of these observations, we note a number of strengths. First, we followed recommendations for best practices in cognitive pupillometry (Mathôt and Vilotijević, 2023) by measuring the pupil response to the same constant stimulus (the onset-probability signal), at the same central location, in a slow-moving experiment. Second, the sample sizes in our second (*n* = 39) and third (*n* = 25) experiments are large relative to others reported in the literature (e.g., 12 and 15, respectively, for the two empirical studies cited in the next paragraph). Third, we applied a variety of statistical techniques (e.g., bootstrapped 95% confidence intervals, ROI-based *t* tests, and linear mixed-effect modeling of the trial-level data with cross-validation) to ensure that our results did not hinge on any one particular approach. Fourth, our primary finding from Experiment 1 (that vertical onset-probability signals generated smaller pupil areas than horizontal signals) was independently replicated in Experiment 2, using a sample size that was more than double that used in Experiment 1.

Future work is needed to work out a mechanistic explanation of these results. One speculative possibility involves the preallocation of covert endogenous spatial attention to task-relevant locations. In Experiments 1 and 2, the target emoji and its scrambled counterpart always appear at fixed locations along the horizontal meridian. Thus, participants may preallocate covert attention to these two locations to aid in the localization task, especially as the opacity of the images drops. When the onset-probability signal is horizontal, it aligns with, and symbolically “points to,” each of the relevant locations along the horizontal meridian. When the onset-probability signal is vertical, it aligns with task-irrelevant locations along the vertical meridian. If covert spatial attention is allocated earlier in time (i.e., during the presentation of the onset-probability signal) when the signal aligns with task-relevant locations, then covert spatial attention would be allocated to relatively dark regions of the display, where task stimuli have not yet appeared. Converging evidence from Binda and colleagues, as well as Mathôt and colleagues, has shown that covertly attending to a dark surface, relative to a bright surface, leads to a larger pupil (Mathôt et al., 2013; Binda et al., 2014). Taken together, allocating covert spatial attention to a dark surface when the onset-probability signal is horizontal but not vertical would generate the pattern of results reported in Experiments 1 and 2. This hypothesis does not perfectly explain the results from Experiment 3, as we do not see a complete reversal of the orientation effect, only a significant weakening. That said, the idea that alignment/misalignment of the orientation of the onset-probability signal and the task locations might induce trial-to-trial differences in covert attentional strategies, which would then modulate the pupil area, is a plausible starting point for future work.

Whatever the mechanistic explanation may turn out to be, the significance of this work lies more in its usefulness as a methodological “cautionary tale” than in revealing a new insight into pupillary function. Perfect luminance matching, achieved by using the same task-irrelevant physical stimulus (a simple, white line) in two different orientations, was not enough to prevent inducing an unwanted methodological complication. We had no a priori reason to suspect that a physically identical onset-probability signal, presented in two different orientations, would modulate the pupil area in the experimental design specific to Experiments 1 and 2. In a bit of bad luck, likely due to the small sample size, we misinterpreted preliminary results from Experiment 1 as an effect of onset-probability information. It was only after expending time and money on a replication and extension attempt that we discovered those results were better explained by a complex interaction between the orientation of the onset-probability signal and the task-relevant locations.

In closing, we underscore an important point. Whenever an experiment manipulates stimuli between conditions by changing some physical aspect (e.g., orientation), a confounding pupillary effect is always possible. That said, the complex effect observed in our study is not necessarily generalizable to other pupillometry work. As demonstrated in Experiment 3, the orientation effect we reported can be extinguished simply by relocating the task-relevant positions, making it unlikely to directly compromise other studies that typically involve even greater visual variation from that used in our Experiments 1 and 2. Nevertheless, our findings demonstrate that seemingly inconspicuous visual features can, under certain circumstances, exert strong and unforeseeable influences on the pupil area. Taken together, our findings underscore the remarkable sensitivity of the pupil to subtle visual changes, highlighting both the importance of cautious experimental designs and promising new directions this sensitivity may offer the field of cognitive pupillometry.

## Synthesis

Reviewing Editor: Frederike Beyer, Queen Mary University of London Decisions are customarily a result of the Reviewing Editor and the peer reviewers coming together and discussing their recommendations until a consensus is reached. When revisions are invited, a fact-based synthesis statement explaining their decision and outlining what is needed to prepare a revision will be listed below. The following reviewer(s) agreed to reveal their identity: Paola Binda.

Both reviewers highlighted the interest of your findings to the field and generally supported the analyses run and the conclusions drawn. As you can see, however, they also raised some important confounds and missing controls which should be addressed and discussed.

To fully support the current conclusions, additional data collection with an appropriate control condition might be ideal. That said, the reviewers and I agreed that the current data are sufficient for publication as post-hoc findings with appropriate discussion of potential confounds. To enhance the strength of the current findings, please consider the suggested control analyses, such as comparing to a pre-signal baseline and controlling for potential differences in fixation and for multiple comparisons.

Reviewer comments:

The paper investigates whether expectation about a peripheral event modulates pupil size, and reports a secondary, unexpected finding that stimulus orientation-despite being luminance-matched and task-irrelevant-systematically influenced pupil constriction. The main claim is that even when luminance is controlled, orientation can act as a confound in cognitive pupillometry.

This is a potentially important methodological observation, but the paper is currently limited in interpretation, analysis, and control of alternative explanations. The points below summarise key concerns and suggestions.

1) The Introduction implies that expectation might cause anticipatory constriction, which could in turn enhance processing of low-probability cues. This mechanistic chain should be more clearly unpacked. The authors should also consider prior work linking expectation and prediction error to pupil size (e.g., Nassar et al., 2012; Kafkas, 2021).

2) The orientation effect is presented as surprising, but prior research has shown that orientation and shape can influence pupil size via perceptual or attentional mechanisms. The authors should acknowledge this literature and clarify whether their finding aligns with or contradicts previous results (e.g., Laeng &Endestad, 2012; Sasaki et al., 2006).

3) The emergence of the orientation effect in the second half of the session may reflect learning or habituation rather than contextual congruency. Participants may have implicitly associated the signal with task structure despite instructions. This possibility undermines the claim that the orientation effect reflects a purely low-level or automatic influence on pupil size.

4) All experiments lack a true baseline condition without an orientation signal. This limits interpretation, as the comparison is always relative (vertical vs horizontal). A neutral baseline (e.g., fixation-only) would help determine whether vertical lines induce increased constriction, horizontal lines produce dilation, or both.

5) The 500 ms region of interest (ROI) prior to onset is reasonable, but may still capture preparatory activity tied to upcoming events. A comparison to a pre-signal baseline would help disambiguate effects specific to orientation or probability cues.

6) The authors rely on linear interpolation during blinks but do not report how many samples were interpolated or how many trials were excluded. This is of relevance given the small magnitude of the reported effects.

7) Pupil size may vary with gaze angle. Although a chin rest was used, the authors should report gaze stability across conditions using eye tracker data, to rule out fixation-related confounds.

8) Bootstrapping and time-series analysis add rigour, but the potential for inflated false positives due to multiple comparisons and arbitrary ROI selection is not addressed. Correction for multiple comparisons should be explicitly reported.

9) The claim that vertical lines are incongruent with horizontal task layout presumes that participants process the line as directional or spatially meaningful. This assumption is not clearly supported, and congruency alone may not explain the effect.

10) The peripheral flashes may have elicited covert attentional shifts despite being task-irrelevant. This introduces the possibility that expectation effects on pupil size were masked or diluted by involuntary attention shifts, especially in Experiment 2.

11) Orientation-specific processing in early visual cortex may also contribute. Vertical and horizontal lines differ in spatial frequency content and cortical representation, which could differentially engage the visual system and modulate pupil constriction independently of attention.

12) Perceptual fluency or familiarity may offer another explanation. Horizontal lines are more prevalent in natural and cultural environments (e.g., horizon, text), potentially affecting perceptual processing and pupil responses.

13) The dissociation between orientation and expectation effects remains unclear. While cue-probability contingencies were counterbalanced, the statistical power to detect orientation-by-expectation interactions may have been limited. A formal test (e.g., mixed-effects modelling with interaction terms) would clarify whether orientation fully accounts for effects initially attributed to expectation.

14) It is uncertain whether the reported orientation effect generalises. The stimuli were centrally presented; it remains to be tested whether similar effects would occur with peripheral cues or different display configurations.

15) The Discussion appropriately emphasises methodological caution for pupillometry but stops short of addressing implications for broader cueing paradigms. Orientation may implicitly bias attention or expectation even when nominally task irrelevant.

16) Related to the broader caution that pupillometry may be influenced by orientation as well as luminance, the manuscript does not offer concrete guidance on how such confounds might be controlled. Do the authors intend to suggest that most pupillometry studies are potentially compromised, or do they have specific recommendations for mitigating orientation-related effects in future work?

The manuscript describes a novel phenomenon, a pupil-size modulation reflecting the contingency between the orientation of a central cue and the location of task-relevant stimuli.

I have no concerns with the experimental approach or the analysis plan, both of which are rigorous and clearly presented.

I only have minor suggestions for the way the story is framed:

- there is no strong evidence that pupil-size changes (especially the minuscule ones that are relevant here) produce any measurable impact on visual performance. the introduction uses this as a starting point, referencing two published papers that suggest such link between pupil size and visual performance. however, this evidence is far from direct, being entirely predicated upon correlations (which is not causation!). for this reason, I would recommend rephrasing the beginning of the introduction - this point is marginally relevant for the reported study anyway.

- while I agree that the emphasis should not be on the mechanistic explanation of the reported phenomenon, I see another reason why this is important besides avoiding confounds in future research. It shows that the sensitivity of the pupillometry technique to subtle features of the task structure is exquisite. I would encourage the authors to present their results under this more positive light (rather than adding orientation to the list of the confounds that one needs to worry about when measuring pupil size).
